# HPV detection in the oral and genital mucosa of women with positive histopathological exam for genital HPV, by means of the PCR

**DOI:** 10.1016/S1808-8694(15)30773-4

**Published:** 2015-10-19

**Authors:** Therezita M. Peixoto Patury Galvão Castro, Ivo Bussoloti Filho, Velber Xavier Nascimento, Sandra Doria Xavier

**Affiliations:** 1 Master's degree in medicine, Faculdade de Ciências Médicas da Santa Casa de São Paulo. Graduate doctoral student, Faculdade de Ciências Médicas da Santa Casa de São Paulo; 2 Doctorate in medicine, UNIFESP. Adjunct professor of Otorhinolaryngology, Faculdade de Ciências Médicas da Santa Casa de São Paulo, FCMSCSP; 3 Graduate doctoral student in molecular biology; 4 Master's degree in Otorhinolaryngology, Faculdade de Ciências Médicas da Santa Casa de São Paulo

**Keywords:** oral cavity, genitalia, papillomavirus

## Abstract

Infection by the Human Papilloma Virus (HPV) is one of the most frequent sexually transmitted diseases all over the world. The relationship between oral and genital HPV remains uncertain, as it is with its role on oral carcinogenesis. The goal of the present investigation was to check for the presence of HPV DNA in the oral and genital mucosas of women with HPV genital infection, using the polymerase chain reaction (PCR).

**Study method:**

Cross-sectional cohort.

**Materials and Methods:**

this is a pilot and prospective study involving 30 women, aged between 14 and 51 years, with HPV genital infection, confirmed by histopathology. All the patients were submitted to the exam and sample collection by swabbing the oral and genital mucosas in order to test for HPV DNA through the PCR technique.

**Results:**

none of the oral cavity samples were positive for HPV, while in the genital tract, HPV was detected in 17 (57%) of the 30 patients, especially HPVs 6b and 16.

**Conclusion:**

Results show a higher percentage of genital HPV in comparison to the oral cavity, and suggest that genital HPV does not seem to be a predisposing factor for the oral infection in the same patient.

## INTRODUCTION

The human papillomavirus (HPV) belongs to a heterogeneous^1^ group of DNA viruses that infect the skin and mucosae in various sites of the human body.^2^

The transmission mode of this virus to the oral mucosa is not clearly known. It has been suggested that transmission may occur during vaginal birth, self-inoculation, or oral sex.^3^, ^4^, ^5^

It appears that saliva has a protective role against HPV infection due to the presence of antimicrobial agents such as lysozymes, lactoferrin, IgA and cytokines.^6^^,^^7^

The most frequently affected sited in the mouth are the lips, palate, tongue, gingiva, uvula, tonsils, and the floor of the mouth. Saliva accumulates on the floor of the mouth; cancerous agents dissolved in saliva, such as tobacco and alcohol, provide ample opportunity for harmful viral activity.^8^^,^^9^

HPV infection has classically been divided into three separate manifestations: clinical, subclinical and latent forms. Clinical infection is easily detected with the naked eye, in the form of a wart. The subclinical form occurs more frequently on the cervix (80% of cases) and is diagnosed by colposcopy after applying 5% acetic acid. The latent form is diagnosed only by molecular biological tests.^10^^,^^11^

Biopsies are used to study the pathology of a specimen and to grade the disease. Molecular biological tests are required for diagnosing HPV and defining its type.^12^

Hybridization tests of tissue samples and smears are currently the methods of choice for detecting the HPV DNA. These methods include the dot blot, the Southern blot, in situ hybridization, and the polymerase chain reaction (PCR); the latter is the most sensitive of these tests.^13^^,^^14^

The PCR increases–by many million times–minute amounts of target DNA sequences. Initiating systems (primers) are used, the most common being the MY09-MY11 and the GP5-GP6 primer systems.^15^, ^16^, ^17^

HPV types vary according to their tissue tropism, their associations with different lesions and their oncogenic potential. These types may be characterized as low risk HPVs for developing malignancies (types 6, 11, 41, 43, 44) and high risk HPVs for developing malignancies (16, 18, 31, 33, 35, 39, 45, 46, 51, 52). Types 6 and 11 are the two main types involved in most of the genital warts (condylomas); types 16 and 18 are found mainly in cervical cancer.^12^^,^^16^^,^^17^

Over 100 HPV types have been found to date.^18^, ^19^, ^20^ Of these, 25 types (HPV-1, 2, 3, 4, 6, 7, 10, 11, 13, 16, 18, 31, 32, 33, 35, 40, 45, 52, 55, 57, 58, 59, 69, 72 and 73) have been associated with benign oral lesions (squamous cell papilloma), condylomata acuminata, common warts, focal epithelial hyperplasia, and malignancies such as the squamous cell carcinoma.^21^

Many researchers have studied oral and genital HPV to discover whether genital infection by this virus could lead to infection elsewhere, such as in the mouth.^22^, ^23^, ^24^, ^25^, ^26^

Van Doornum et al.^22^ (1992) applied the PCR to study oral and genital HPV in 65 males and 111 females. Examination of the mouth consisted of unaided visual inspection and scrapes for further study. Genital HPV was found in 21 (32%) of 65 males and 25 (23%) of 111 females; none of these patients had oral HPV. There were no reports of oral sex.

Badarrocco et al.^23^ (1998) applied the PCR and found oral HPV in five of ten women diagnosed with genital HPV. Unaided visual inspection of the mouth and colposcopy was don in all patients; oral scrapes were taken. There were no reports of oral sex.

Camadas et al.^24^ (2004) applied the PCR to study genital and oral HPV in 188 females. Unaided visual inspection was done of the mouth and scrapes were made. The positivity rate in the mouth was 15 (7.9%). There were no reports of oral sex.

Giraldo et al.^25^ (2006) found positive HPV PCR testing in 29 (20.7%) of 140 females with or with no genital lesions. Scrapes were taken from the oral mucosa in which unaided visual inspection had been carried out. These authors also highlighted that genital HPV was present in 26 (89%) of 29 females with oral HPV, as detected by the PCR. Oral sex was practiced by 16 (55.2%) of 29 women with positive oral HPV, and by 59 (23.2%) of 111 women that tested negative for oral HPV.

Xavier et al.^26^ (2007) applied the PCR to test 10 males with PCR-confirmed genital HPV for oral HPV. Unaided visual inspection was done of the mouth and scrapes were made. None of these patients had the HPV in the oral mucosa; there were no reports of oral sex.

Further investigation is required on this theme, considering the conflicting results described in the literature about the presence and relation of oral HPV with genital HPV.

The purpose of this study was to verify the presence of HPV DNA in the oral and genital mucosa of patients with genital HPV infection by applying the PCR technique.

## MATERIAL AND METHOD

A retrospective study was done from May 2005 to November 2006. The Research Ethics Committee of the UFAL approved the study (protocol number 005564/02-50). All patients were informed about the purpose of the study and the required procedures if participating. All of them read and signed a free informed consent form.

The inclusion criteria were sexually active female subjects aged from 14 to 51 years that presented clinical or subclinical genital HPV. Exclusion criteria were patients with immunological conditions, such as HIV infection, immunosuppressed patients, and users of immunosuppressant drugs.

Thirty patients with genital warts (clinical and/or subclinical) suggesting HPV infection were recruited. All subjects underwent a gynecological exam, colposcopy (with 5% acetic acid), and biopsies of warts for diagnosis. Scrapes for cytology using sterilized brushes (colpocytology kits, Libbs®) were taken from genital lesion sites (external genital, vagina and cervix) during colposcopy.

On the same day, unaided visual inspection was made of the oral cavity, using artificial light (photophore) in an attempt to identify any clinical lesion that might suggest HPV infection. Samples were taken from sites where the HPV was likely to be found by scraping the oral mucosa with sterilized brushes (the soft palate, the uvula, the tonsils, dorsum of tongue, the sublingual area, and the jugal mucosa. Examination of the mouth did not include use of a colposcopy or application of acetic acid.

The scrapes were placed in separate vials containing a buffer solution (tris-HCL 10mM, pH 8, EDTA 1mM) and identified as either genital or oral; the vials were sent to the Molecular Biology Laboratory of the Universidade Federal de Alagoas (UFAL) and stored at −20°C for testing with the PCR technique.

Extraction of genomic DNA was done using the GFX kit (Asmersham Bioscience) method; the DNA concentration in each sample was estimated using a spectrophotometer (Genova). The MY09/MY11 primer systems were used for viral DNA amplification; viral types were identified according to their electrophoretic patterns.

Subjects also answered a standard questionnaire on epidemiological issues, such as the beginning of sexual life, the number of partners, oral sex, use of alcohol and smoking.

## RESULTS

The epidemiological survey revealed that 22 of 30 patients (73%) engaged in oral sex, and 16 patients (53%) engaged in anal sex; nine patients (30%) were smokers and none consumed alcoholic beverages; Table 1 presents these results.

**Table 1:** Individual results of the 30 subjects in this study

N	Age	Genital lesion	Site	HPV type	Histological diagnosis	Beginning of sexual activity	Number of sexual partners	Oral sex	Anal sex	Smoking
1	32	subclinical	cervix	16	SIL high grade	17	3	no	no	no
2	30	subclinical	Cervix	16	Alt. suggest. HPV	17	4	yes	yes	yes
3	36	Clinical / subclinical	Cervix / vulva	6b	SIL low grade	17	2	yes	yes	no
4	19	subclinical	cervix	–	Alt. suggest. HPV	13	1	yes	no	no
5	24	subclinical	cervix	In.	SIL low grade	22	1	no	no	no
6	29	subclinical	Cervix, vagina	–	Alt. suggest. HPV	19	4	yes	yes	no
7	39	subclinical	cervix	–	SIL low grade	17	5	yes	no	no
8	21	Clinical	Cervix, vulva, vagina	6b	condyloma	20	1	no	no	no
9	15	Clinical / subclinical	Cervix / vulva	–	Alt. suggest. HPV	14	1	yes	no	no
10	21	subclinical	cervix	–	SIL low grade	18	5	yes	yes	no
11	51	subclinical	cervix	66	SIL low grade	22	3	yes	yes	no
12	42	subclinical	cervix	–	Alt. suggest. HPV	15	2	yes	yes	yes
13	21	Clinical / subclinical	Cervix, vulva, vagina	6b	SIL low grade	16	2	yes	yes	yes
14	19	subclinical	Vulva, vagina	–	Alt. suggest. HPV	15	2	yes	yes	yes
15	17	Clinical / subclinical	Cervix, vulva	6b	condyloma	12	2	yes	no	no
16	36	subclinical	cervix	–	Alt. suggest. HPV	18	2	yes	no	yes
17	25	subclinical	cervix	16 e MM8	SIL low grade	13	3	yes	no	no
18	32	subclinical	vagina	–	Alt. suggest. HPV	15	6	yes	yes	no
19	45	Clinical	Vulva, anus	6b	condyloma	19	4	yes	yes	yes
20	32	subclinical	cervix	In.	SIL low grade	17	1	no	no	yes
21	19	subclinical	Cervix, vagina	–	SIL low grade	15	3	yes	yes	no
22	14	Clinical / subclinical	Cervix, vulva, vagina	16	SIL high grade	14	1	yes	yes	no
23	32	subclinical	cervix	31	SIL low grade	16	2	yes	yes	no
24	21	Clinical / subclinical	Cervix, vulva	6b	Alt. suggest. HPV	15	2	yes	yes	no
25	24	subclinical	cervix	6b	SIL low grade	15	1	yes	yes	no
26	16	Clinical / subclinical	Cervix, vulva / vagina	–	Alt. suggest. HPV	13	4	yes	yes	yes
27	32	subclinical	Cervix	52	SIL low grade	15	1	no	no	no
28	36	Clinical / subclinical	Cervix, vulva / vagina	–	Alt. suggest. HPV	28	2	no	no	no
29	34	Clinical	vagina	In.	Alt. suggest. HPV	18	1	no	no	no
30	32	subclinical	cervix	–	SIL high grade	22	2	no	no	no

HPV=human papilloma virus; In=Indeterminate; SIL=Squamous intraepithelial lesions

The results of colposcopy showed subclinical lesions in 19 patients (63%) of, subclinical lesions in eight patients (27%), and clinical lesions in three patients (10%), located mostly on the cervix. All 30 samples of genital lesions were positive for HPV. Table 1 shows these results.

The results of PCR testing of genital samples were positive in 17 cases (57%) of the 30 patients; 13 cases had negative samples. The most frequent HPV type was the 6b type, found in seven (41%) of the 17 positive samples, followed by type 16 in four samples (23%). Types HPV66, HPV52, and HPV31 were found in one sample each (6%). The type could not be determined in three samples (18%).

Table 1 shows these results.

Unaided visual inspection of the mouth of 30 subjects revealed no clinical lesions suggesting HPV.

PCR test results in 30 samples of oral scrapings were HPV negative.

## DISCUSSION

In this study 19 (63%) of 30 patients had only subclinical genital HPV infection, mostly in the cervix, which was confirmed by colposcopy. This is similar to other published studies, showing that this is the most frequent HPV infection.^10^^,^^11^

Histology is an important method for diagnosing HPV infection; all of the genital samples had histological changes that suggested this diagnosis. This may be considered as a routine first exam for the diagnosis of HPV infection. However, since histology is unable to identify the HPV, molecular biology techniques are needed for confirming HPV infection;12 this is done mostly using PCR techniques, which is more sensitive for detecting the HPV.^13^^,^^14^

The PCR technique was the method we chose to identify oral and genital HPV in this study; we used the MY09-MY11 primer systems,^16^, ^17^, ^18^ which is the most sensitive^13^^,^^14^ and preferred method for detecting HPV DNA in tissue smears and samples.^14^

The most frequent HPV types found in the genital area of 17 patients that were PCR-positive for HPV were the types 6b (41%) and 16 (23%). The former is a low-risk type and is frequently found in genital condylomas; the latter is found mainly in cervical cancer.^17^

The number of smokers in the 30-patient sample was nine (30%); none of them consumed alcoholic beverages. These variables did not affect the results, although they are factors that favor HPV infection in the mouth.^8^^,^^9^

The samples of the mouth were PCR-negative in all 30 patients of this study. In other studyes^22^, ^23^, ^24^, ^25^, ^26^ that used the same technique, the positivity rate ranged from 0 to 50%. We concluded that the presence of genital HPV infection does not appear to be a predisposing factor for oral HPV infection. This result is important for patients with genital HPV infection who become worried about possible transmission of the disease to their partner(s) and to other bodily sites.

Low immunological defense is probably the most important factor for HPV infection in other sites of the body. The mouth contains IgA and proteolytic enzymes that protect against HPV infection.^6^^,^^7^

HPV transmission to the mouth is not yet clearly understood.^3^, ^4^, ^5^ The rate of oral sex was high in our sample; however, the results of oral HPV infection were negative in 73% of cases. Compared with other studies,^22^, ^23^, ^24^, ^25^, ^26^ we found that few patients were aware of this transmission mode to the mouth; it was thus impossible to conclude whether patients with genital lesions that engaged in oral sex were more predisposed to have oral HPV infection.

Further studies are needed on the relation between oral and genital HPV infection to explain its action on the oral mucosa.

## CONCLUSION

In this study, the percentage of HPV infection was higher in the genital area (57%) compared to the oral mucosa (0%); this suggests that genital HPV is not a predisposing factor for oral HPV infection in the same patient.
